# Multiblock copolymers of PPC with oligomeric PBS: with low brittle–toughness transition temperature†

**DOI:** 10.1039/c8ra01588k

**Published:** 2018-04-18

**Authors:** Jiaxiang Qin, Limiao Lin, Shuanjin Wang, Shuxian Ye, Weikeng Luo, Min Xiao, Dongmei Han, Yuezhong Meng

**Affiliations:** The Key Laboratory of Low-carbon Chemistry & Energy Conservation of Guangdong Province, State Key Laboratory of Optoelectronic Materials and Technologies, School of Materials Science and Engineering, Sun Yat-sen University Guangzhou 510275 PR China mengyzh@mail.sysu.edu.cn wangshj@mail.sysu.edu.cn

## Abstract

In order to decrease the brittle–toughness transition temperature and increase the mechanical strength of poly(propylene carbonate) (PPC), a series of multiblock copolymers of poly(propylene carbonate)-*multiblock*-poly(butylene succinate) (PPC-*mb*-PBS) are designed and synthesized. ^1^H-NMR, DOSY and GPC results demonstrate the successful synthesis of PPC-*mb*-PBSs with designed multiblock sequence. The thermal, crystalline and mechanical properties of these PPC-*mb*-PBSs are evaluated by DSC, TGA, POM, tensile and tearing testing. Experiment results demonstrate that crystallinity, thermal and mechanical properties of PPC-*mb*-PBSs can be readily modulated by changing the composition and block length of PPC and PBS moieties. It is found that all the prepared PPC-*mb*-PBSs are semi-crystalline polymers with a melting temperature at 93–109 °C and a *T*_g_ at around −40 °C. Both crystallization rate and crystallinity of the multiblock copolymers increase with increasing both PBS content and PBS block length. As a consequent, the tensile strength increases with increasing PBS/PPC block ratios at room and lower temperatures. In conclusion, the amorphous PBS phase in the block copolymers acts as soft segment, endowing PPC-*mb*-PBS copolymers with much better flexibility than PPC at low temperature of 273 K when PPC segments are frozen.

## Abstract 2

In present work, biodegradable multiblock copolymers from oligomeric PPC and PBS with low brittle–toughness transition temperature and superior mechanical properties was synthesized, making it more potential candidate as packaging materials.

## Graphical abstract

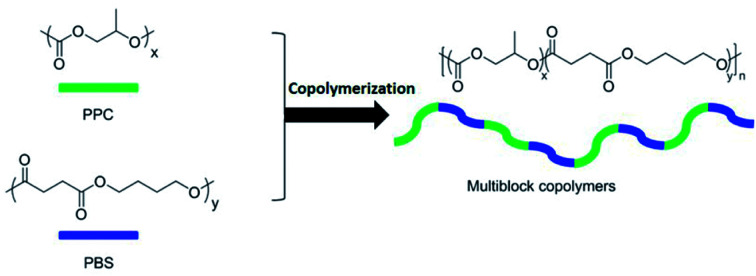

## Introduction

Aliphatic polyesters are known biodegradable polymers with good biocompatibility, biodegradation, low immunogenicity and non-cytotoxicity, and have been introduced as integral components of *e.g.* engineered tissues, medical devices, and drug delivery systems.^1–9^ Currently, there is increasing interest in functional poly(propylene carbonate) that can be prepared directly from carbon dioxide (CO_2_) and propylene oxide (PO), represent a promising class of biodegradable polymers for a variety of applications.^10–13^ However, due to the low *T*_g_ and the amorphous nature, PPC shows highly softness and low mechanical strength at a temperature slightly higher than room temperature.^14^ Particularly, PPC exhibits a very brittle nature in case it is used in the application of a film material.^15–17^ In this senses, the application of PPC has been severely restricted as thermal plastic.^18^ To address these issues, numerous studies have been proposed to improve the properties of PPC. One effective approach is to introduce bulky or hard segment into the backbone of PPC *via* copolymerization process.^19–21^ Recently, the researches focus on linking a number of distinct homopolymers *via* covalent bonds to provide di- and triblock copolymers.^22–27^ A number of typical monomers have been used to synthesize polycarbonate-*block*-polyester polymers with CO_2_, such as lactide,^22,23^ cyclohexene oxide (CHO)^24^ as well as cyclic acid anhydride.^25–27^ Unfortunately, these strategies are only suitable for CHO and cyclic anhydride monomers, since few monomers are more reactive for copolymerization with PO and CO_2_.^23,28^ The mechanical properties of copolymers could be enhanced by copolymerizing PPC with lactide. However, due to the small size of lactide, such random, di- and triblock copolymers have a limitation in free volume, leading to no significant improvement in the brittle–toughness transition temperature.

On the other hand, biodegradable polymers with a multiblock topology can be facilely prepared by coupling reaction.^29–40^ Through the coupling reaction, the physico-chemical properties could be strongly complemented and easily controlled by varying the block length and total molecular weight of the polymer. Many biodegradable multiblock copolymers based on poly(butylene succinate) (PBS),^29,30^ poly(lactide),^33–35,38,39^ polycaprolactone,^38,39^ poly(hydroxybutyrate),^40^ and poly-(ethylene glycol)^34,35,40^ have been prepared to improve their physicochemical properties for biomaterials applications.

PBS synthesized from succinic acid (SA) and 1,4-butanediol (BDO) is a commercially available, aliphatic thermoplastic polyester with many interesting properties, including biodegradability, mechanical properties, melt processability, and thermal resistance, thus being a promising polymer for various potential applications.^32–34^ In our previous work, blends of PPC and PBS were prepared to improve the thermal and mechanical properties of PPC.^41,42^ However, as far as we know, there is no report on the multiblock copolymer of biodegradable polyester from PPC and PBS.

In this paper, we design and report the synthesis of a series of PPC-*mb*-PBSs from dihydroxylated PPC (PPC–OH) and dicarboxylated PBS (PBS–COOH) *via* a coupling reaction. A carefully control over the terminal functions of the prepolymers makes it possible to directly adjust 1 : 1 molar ratio between two different functional groups, which results in the easy formation of high-molecular weight PPC-*mb*-PBSs. The multiblock copolymers show various improved properties, including processability, mechanical strength, especially low brittle–toughness transition temperature.

## Experimental

### Materials

Propylene oxide (PO, AR), 1,4-butanediol (BDO, AR), succinic acid (SA, AR), tetrabutyl titanate, dimethylaminopyridine (DMAP, AR) and *N*,*N*′-dicyclohexylcarbodiimide (DCC,AR) was purchased from Aladdin chemicals Co., Ltd. Neat PPC with high molecular weight was supplied by Henan Tianguan Group while neat PBS (Bionolle #1020, *M*_n_ = 100 kDa) purchased from Showa Highpolymer Co., Ltd. BDO, SA, DMAP, DCC, tetrabutyl titanate and all the solvent was used as received.

### Synthesis of high molecular weight of PPC

PO was refluxed over CaH_2_ and distilled under dry nitrogen gas flow. CO_2_ of 99.99% purity was used as received. Zinc glutarate were prepared according to previous work.^43^ PPC were prepared by copolymerization of CO_2_ and PO using zinc carboxylates as catalyst. Typically, 0.5 g catalyst and 50 mL PO were introduced into the cleaned autoclave, pressured to 5 MPa with CO_2_ and maintain at 60 °C for 40 h. The resulting polymers were dissolved in chloroform and then precipitated by being poured into vigorously stirred ethanol. The final product was filtered and dried under vacuum at a temperature of 100 °C for 24 h.

### Preparation of PPC–OH

BDO (0.57 g, 7.5 mmol) and high molecular weight PPC synthesized from last step (5.1 g, 50 mmol) were added to a three-necked flask (25 mL), which was connected to a manifold equipped with vacuum and N_2_ gas lines, and equipped with a mechanical stirrer. The flask was made oxygen-and moisture-free by a nitrogen purge followed by use of a vacuum before was immersed in a hot-oil bath (180 °C) and the reaction lasted for 120 min. After cooling to room temperature under the inert atmosphere, the resultant PPC–OH was washed by 5% hydrochloric acid and water 3 times to remove the residual catalyst.

### Preparation of PBS–COOH

SA (38.9 g, 0.3 mol) and BDO (32.4 g, 0.3 mol) were added into a 150 mL three-necked round-bottom flask, which was connected with gas inlet and outlet adapters. The mixture was immersed in a silicon oil bath and then reacted at 160 °C under nitrogen atmosphere. It is well known that the polymerization consisted of esterification and polymerization reactions; in the process of esterification, H_2_O (0.6 mol) was collected using a trap device and the reaction was completed for about 3 h. Then appropriate amount of catalyst tetrabutyl titanate was added to the system and the viscous liquid was heated to 220 °C to undergo first-stage polymerization at 2.0 torr for 2 h with the removal of water generated. At the end of the first-stage polymerization, the pressure in the reaction vessel was reduced to 0.5 torr, and then the second stage polymerization proceeded for 2–6 h to further increase the molecular weight of the resulting linear PBS. After the polymerization was completed, the temperature in the reaction vessel was cooled down to 160 °C and then appropriate amount of SA was added again into the system. The PBS polymer was then reacted with the added SA at 160 °C for 120 min under nitrogen atmosphere. After the end-capping reaction, about 100 mL of chloroform were used to dissolve the product. The solution was precipitated into excessive ethanol to obtain a white powdery polymer. The filtered polymer was washed with ethanol and dried at 25 °C for 24 h in a vacuum oven.

### Synthesis of PPC-*mb*-PBSs multiblock copolymers

PPC–OH (1 mmol) and equimolar dicarboxylated PBS were dissolved in 50 mL of anhydrous methylene chloride. DMAP (0.3 mmol) was added to the solution as a catalyst and DCC (3 mmol) was added as a coupling agent. The reaction flask was kept in a dry nitrogen environment at room temperature while stirring. After 10 min, a white dicyclohexylurea (DCU) precipitate formed as a reaction by-product. Reaction conditions were maintained for 48 h. The precipitated DCU was filtered off. The filtrate was concentrated under reduced pressure and then poured into a large excess amount of cold ethanol with vigorous stirring. The precipitate product was collected and dried in vacuum oven for 24 h. The various multiblock copolymers with different compositions were prepared using the same procedure.

### Film preparation

Prior to measurements of mechanical properties, the film samples of PPC, PBS and PPC-*mb*-PBSs copolymers (thickness ∼ 0.2 mm) were obtained by solution-cast method. The polyester was dissolved in chloroform (10 wt%) and then cast on a polytetrafluoroethylene (PTFE) dish, followed by evaporation of solvent at 25 °C for 24 h. The films were further dried at 40 °C in vacuum for 24 h.

### Measurement

^1^H-NMR spectra of the polyesters were recorded on a Bruker DRX-500 NMR spectrometer at room temperature. Deuterated chloroform (CDCl_3_) was used as solvent, chemical shifts were expressed in ppm with respect to tetramethylsilane (TMS). Diffusion ordered spectroscopy (DOSY) experiments were performed with a Bruker DRX-600 NMR spectrometer operating at 600 MHz, using CDCl_3_ as solvent. Number-average molecular mass (*M*_n_)and polydispersity index (PDI) of the resultant polymer product were measured using a gel permeation chromatography (GPC) system (Waters 515 HPLC Pump, Waters 2414 detector) with a set of three columns (Waters Styragel 500, 10 000, and 100 000 A) and chloroform (HPLC grade) as eluent. The GPC system was calibrated by a series of poly-styrene standards with polydispersities of 1.02 standards. TGA measurements were performed in a PerkinElmer Pyris Diamond TG/DTA analyzer under a protective nitrogen atmosphere. The temperature ranged from 50 to 500 °C with a heating rate of 10 °C min^−1^. The glass transition temperature (*T*_g_) of the copolymers was measured by a DSC (Netzsch Model 204) and the measurements were carried out under nitrogen flow from −70 to 180 °C at a heating rate of 5 °C min^−1^. *T*_g_ of the samples was determined from the second run. The crystallization morphologies of PBS, PPC and their copolymers were studied using a polarized optical microscope (POM) (ZEISS, Axio Scope A1) equipped with a temperature controller (Linkam THMS 600). Dynamic mechanical analysis (DMA) (Netzsch Model 242) was carried out with a tension mode at 1 Hz, 3 N of static force and 3 °C min^−1^ from −60 to 70 °C. MALDI-TOF spectra were recorded on a Bruker ultrafleXtreme MALDI-TOF spectrometer, with DHB as matrix and THF as solution. The tensile tests were performed using a temperature-controlled tensile tester (New SANS, Shenzhen, China) at 0 and 25 °C with a crosshead speed of 50 mm min^−1^. Five specimens of each sample were tested, and the average results were recorded. The Graves tear strength of the films was measured according to ASTM D 1004 with the same crosshead speed.

## Results and discussion

### Synthesis and characterization

**Scheme 1:**
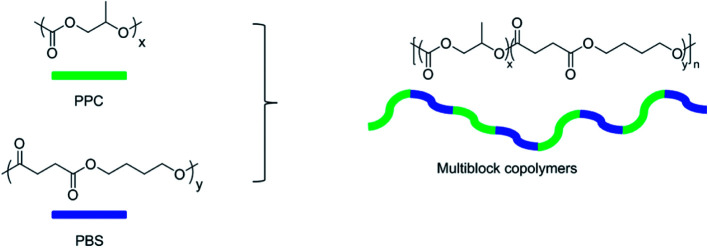
Schematic diagram of PBS-*b*-PPC copolymers.

It has been well documented that the disadvantage of PPC shows relatively low mechanical strength at a temperature around or higher than its *T*_g_ and brittle nature at a temperature lower than its *T*_g_, which restrains its wide applications, especially the application in the area of packaging materials. To address these issues, we introduce oligomeric PBS into the backbone of PPC by the multiblock copolymerization as shown in Scheme 1.

**Scheme 2:**
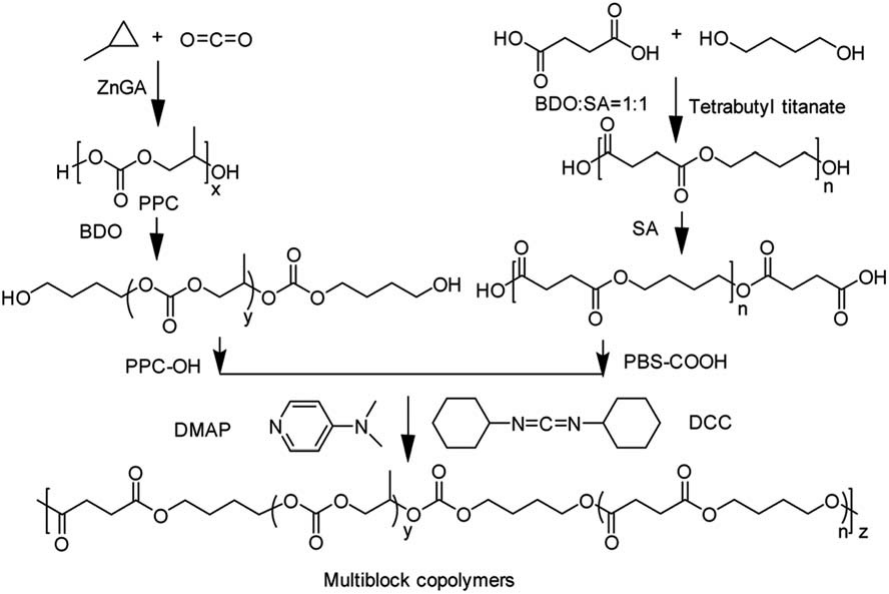
Schematic diagram of synthesis of PBS-*mb*-PPC by coupling reaction.

To obtain linear multiblock copolymers, the prepolymers must be functionalized by reactive end groups for consecutive coupling reactions (Scheme 2). Various dihydroxylated PPC (PPC–OH) with different molecular weight was successfully obtained from alcoholysis of the high molecular weight PPC, while dicarboxylated PBS (PBS–COOH) was synthesized by the esterification of SA and PBS, which was polymerized with BDO and SA in the presence of tetrabutyl titanate. Table 1 lists the molecular weight and its distribution of both PBS–COOH and PPC–OH measured by MALDI-TOF MS. The MALDI-TOF MS patterns were shown in the ESI (Fig. S1 and S2†) indicate the successful synthesis of the hydroxyl-terminated oligomeric PPC and carboxyl-terminated oligomeric PBS.

**Table 1:** Preparation of oligomeric precursors

Oligomer precursor	*M*_n_ × 10^3^	*M*_w_ × 10^3^	PDI
PBS–COOH	1.7	1.9	1.09
	2.7	3.7	1.35
	3.5	5.2	1.44
	4.4	6.4	1.45
PPC–OH	1.2	2.2	1.40
	2.5	3.7	1.38
	3.1	4.3	1.46
	4.0	6.4	1.60

**Fig. 1:**
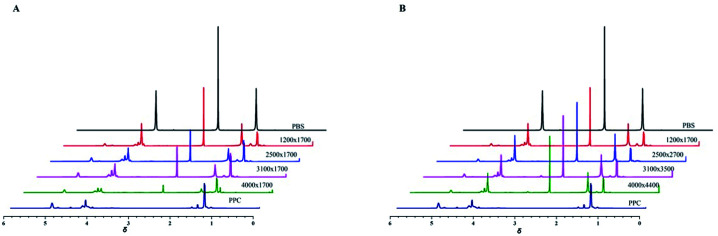
^1^H-NMR spectrum of copolymers: (A) PPC, PBS and PPC-*mb*-PBS with the block size of 1200/1700, 2500/1700, 3100/1700 and 4000/1700; (B) PPC, PBS and PPC-*mb*-PBS with the block size of 1200/1700, 2500/2700, 3100/3500 and 4000/4400.

**Fig. 2:**
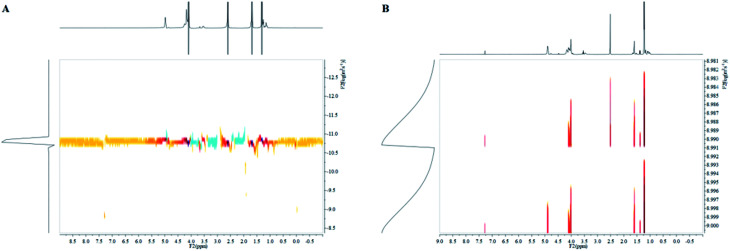
DOSY spectrum of (A) multiblock copolymer and (B) mixture of PPC–OH and PBS–COOH.

**Fig. 3:**
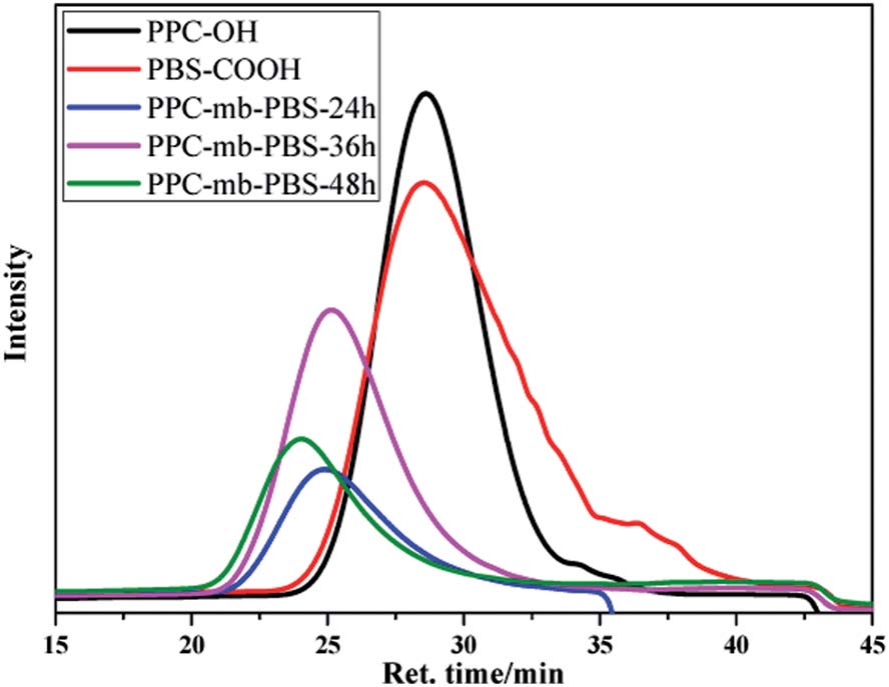
GPC analysis of PPC–OH, PBS–COOH and multiblock copolymer.

A series of PPC-*mb*-PBS multiblock copolymers with different block length (1200/1700, 2500/1700, 3100/1700 4000/1700, 2500/2700, 3100/3500 and 4000/4400) were then synthesized to investigate the effect of block composition and length on brittle–toughness transition temperature and mechanical properties. The prepared PPC-*mb*-PBSs multiblock copolymers were characterized by ^1^H-NMR spectroscopy in CDCl_3_ (Fig. 1). The proton resonances at 1.3, 4.2, and 5.0 ppm correspond to CH_3_, CH_2_ and CH in polycarbonate sequence, while the peaks at 2.7 ppm are attributed to the CH_2_ groups in SA of butylene succinate units. The molar ratio of PPC to PBS segment can be calculated from ^1^H-NMR spectroscopy, which is consistent with theoretical and designed ratio (Table 2). It reveals that PPC and PBS blocks in backbone are multiblock structure. The coupling reaction was also confirmed by DOSY NMR spectroscopy. DOSY NMR is a two dimensional NMR technique, in which the signal decays exponentially due to the self-diffusion behavior of molecules. This leads to two dimensions: the first dimension (F2) accounts for the conventional chemical shift and the second one (F1) for self-diffusion coefficients (*D*). Theoretically, each component of a mixture can be pseudo-separated, based on its own diffusion coefficient on the diffusion dimension.^44^ DOSY can be considered as NMR chromatography because it is a powerful and sensitive NMR tool for analysis of complex mixtures. DOSY NMR of the multiblock copolymer was realized in CDCl_3_ (Fig. 2). The ^1^H-NMR spectrum exhibits signals corresponding to PPC unit (*δ* = 5.0 ppm) and PBS unit (*δ* = 2.7) in the DOSY pattern of both multiblock copolymer and the mixture of PPC–OH and PBS–COOH. However, the diffusion coefficients of the mixture of PPC–OH and PBS–COOH are found to be separated, while that of the multiblock copolymer is found to be a single value, which indicates that the distinct homo-oligomeric PPC and PBS are linked *via* covalent bonds to yield multiblock copolymers. The molecular weight and molecular weight distribution of the multiblock copolymers were further measured by GPC, listed in Table 2. As shown in Fig. 3, GPC trace is observed to be unimodal and moves forward after copolymerization, which further suggests that the obtained copolymer is not a mixture of PPC and PBS but a multiblock copolymer. All these ^1^H-NMR, DOSY and GPC results demonstrate that PPC-*mb*-PBSs with multiblock sequence are successfully incorporated into the backbone of polymer chains.

**Table 2:** Characteristics of PPC-*mb*-PBS

Block length (PPC-*b*-PBS)	Yield/%	Theoretical molar block ratio	Measured molar block ratio^a^	Theoretical weight fraction^b^	Measured weight fraction^c^	*M*_n_ d × 10^4^	*M*_w_ d × 10^4^	PDI^d^
4000 × 1700	74	3.96	5.40	29.8	23.8	3.93	7.72	1.96
3100 × 1700	84	3.08	3.07	35.4	35.5	4.88	8.48	1.78
2500 × 1700	86	2.48	2.58	40.5	39.5	5.18	8.68	1.83
1200 × 1700	88	1.19	1.07	58.6	61.2	2.40	6.80	2.84
2500 × 2700	90	1.56	1.22	51.9	58.0	2.05	3.03	1.48
3100 × 3500	97	1.49	1.28	53.0	56.8	2.15	3.33	1.62
4000 × 4400	94	1.53	1.27	52.4	57.0	2.02	2.77	1.37

a Determined by ^1^H-NMR spectroscopy.

b Theoretical weight fraction of PBS in the PPC-*mb*-PBS.

c Measured weight fraction of PBS in the PPC-*mb*-PBS.

d Measured by GPC.

### Thermal and crystalline properties

**Fig. 4:**
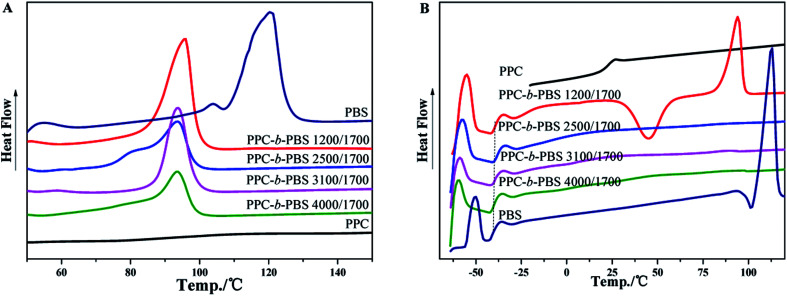
DSC curves of PPC, PBS and PPC-*mb*-PBS with the block size of 1200/1700, 2500/1700, 3100/1700 and 4000/1700: (A) the first heating; (B) the second heating runs.

**Fig. 5:**
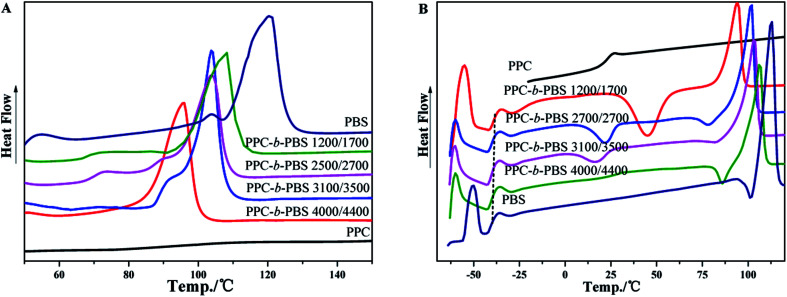
DSC curves of PPC, PBS and PPC-*mb*-PBS with the block size of 1200/1700, 2500/2700, 3100/3500 and 4000/4400: (A) the first heating; (B) the second heating runs.

The thermal properties of PPC-*mb*-PBSs were examined by both DSC and TGA techniques. The melting temperature (*T*_m_), *T*_g_, and crystallinity were summarized in Table 3 on the basis of DSC results. As shown in DSC heating curves, all the multiblock copolymers with various block size give clear melting peak (Fig. 4A and 5A). This is due to the presence of PBS segment which is crystalline in nature. Compared to high molecular weight PBS, the *T*_m_ of the multiblock copolymers decreases with the incorporation of PPC segment. It seems that it is difficult to correlate the *T*_m_ and the length of PPC segment (entry 1, 2, 3 and 4). However, the *T*_m_ of copolymers increases with increasing the length of PBS segment (entry 6–10). Obviously, the melting and crystalline behaviors of PPC-*mb*-PBSs are strongly influenced by the block length of PBS in the multiblock copolymers. According to the heat of fusion obtained from the areas under melting peaks, the crystallinity, *X*_c_, of PBS phase can be calculated from the following formula:
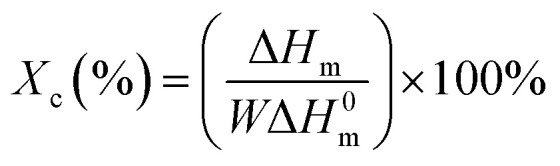
where Δ*H*^0^_m_ is the heat of fusion of 100% crystalline PBS; Δ*H*_m_ is the heat of fusion of the multiblock copolymers, and *W* represents the weight fraction of PBS component in the copolymer. As can be seen in Table 3, the crystallinity of PBS phase is apparently lower in copolymers than in their respective neat PBS, and they slightly decrease with increasing the block length of PPC segment (entry 1–4). This is because that the longer the block length of PPC is, the greater the dimension of amorphous region of PPC is. As a result, due to the confinement and steric effects of PPC, PBS segments are blocked to form crystalline structure by the chain movement, leading to the decrease of crystallinity in multiblock copolymer. From the results of entry 6–9, due to increase of the length of PBS segment, the crystallization ability of PBS in the copolymers is enhanced and led to the increase of the crystallinity of PBS phase in the copolymer.

**Table 3:** Thermal properties of PPC, PBS and copolymers

Entry	Polymer	*T*_m_ a/°C	*T*_g_ a/°C	Δ*H*_m_^a^/J g^−1^	Crystallinity^b^/%	*T*_5%d_ c
1	PPC	—	22.3	—	—	159
2	4000 × 1700	93.6	−39.9	4.95	15.0	226
3	3100 × 1700	93.5	−39.0	8.88	22.7	233
4	2500 × 1700	93.6	−38.1	20.2	27.5	235
5	1200 × 1700	95.9	−39.1	20.8	32.1	270
6	2500 × 2700	104	−40.1	21.5	37.4	261
7	3100 × 3500	104	−40.0	23.5	40.1	242
8	4000 × 4400	108	−40.0	25.7	43.8	259
9	PBS	120	−40.2	52.3	47.3	328

a Measured by DSC.

b Crystallinity was calculated from DSC method (110.5 J g^−1^) for a 100% crystalline PBS.

c Measured by TGA.

In the DSC heating curves of melt-quenched samples (Fig. 4B and 5B), all the multiblock copolymers show the *T*_g_ at −38 to −40 °C, which are comparable with neat PBS. It is believed that this is resulted from the presence of PBS segments in the multiblock copolymer and its miscibility with PPC segments, which will be further confirm by DMA. It should be noted that the *T*_g_ of PPC block appears undetectable in the DSC curves because of its less obviously change in specific heat. Moreover, the clear cold crystallization and melting of PBS blocks are observed in the second heating runs. However, these behaviors are not observed in PPC-*mb*-PBSs with the block size of 2500/1700, 3100/1700 and 4000/1700, indicating no crystallization happen in both melt quenching process and second heating process due to the low content of PBS in these PPC-*mb*-PBSs. There is no cold crystallization peak for the PPC-*mb*-PBS with block size of 4000/4400, but a large melting peak is observed in the second heating curve, indicating the crystallization of PBS segment is completed in the melt quenching process like neat PBS (Fig. S3†). It can be deduced that the crystallization rate of PPC-*mb*-PBSs depends both upon the block ratio of PPC/PBS and block length of PBS. The higher the content of PBS in multiblock copolymer, the higher the crystallization rate is. For the multiblock copolymers with comparable block ratio, the larger the PBS segment, the easier the crystallization is.

Fig. S4 and S5† show the thermal gravimetric analysis results of the multiblock copolymers. The TGA curves of these copolymers clearly indicate two terraces. Apparently, the thermal stability of the multiblock copolymers is greatly improved as compared with PPC because 5 wt% weight lost temperature increases from 158.7 to 258.7 °C (Table 3, entry 5 and 9). The improvement in thermal stabilities is resulted from the introduction of PBS segment. This is because the activation energy of neat PBS decomposition (180 kJ mol^−1^)^45^ is much higher than that of neat PPC decomposition (80 kJ mol^−1^).^46^ As for the degradation behavior of PPC, there are lots of literatures. The most facile decomposition process was an unzipping process by back-biting process forming many cyclic carbonate.^17,47–49^ Therefore, after copolymerization with PBS–COOH, the terminal hydroxyls of PPC were eliminated to a great extent and back-biting reaction can be dramatically restricted. Consequently, the zipper-like depolymerisation reaction of PPC can be greatly prohibited in the presence of PBS segment.

**Fig. 6:**
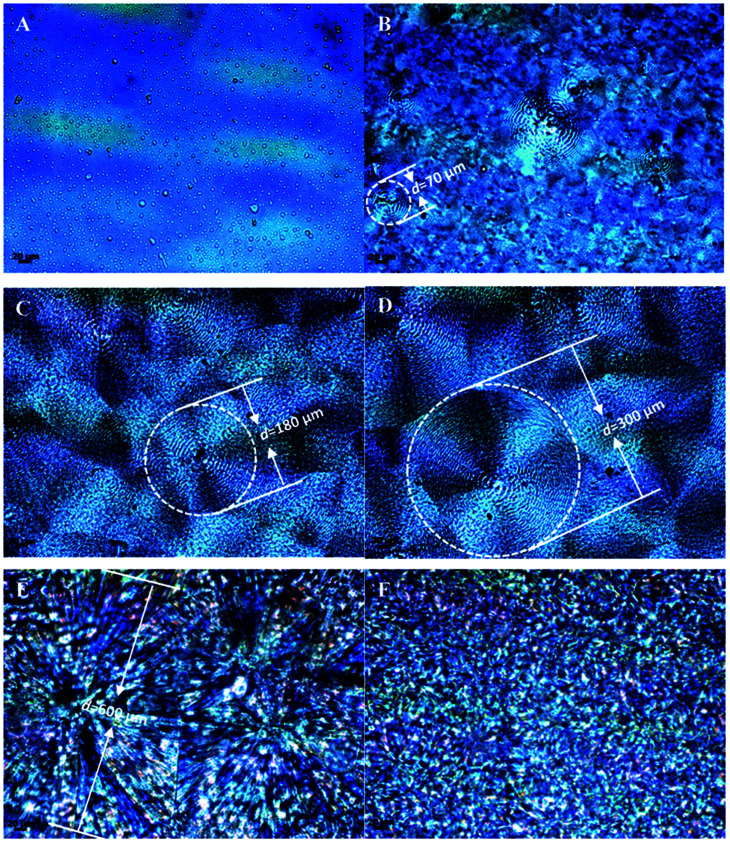
Polarizing optical micrographs of polyester samples crystallized at 90 °C for 2 h. (A) PPC; (B) PPC-*mb*-PBS 1000 × 1000; (C) PPC-*mb*-PBS 2500 × 2700; (D) PPC-*mb*-PBS 3100 × 3500; (E) PPC-*mb*-PBS 4000 × 4400; (F) PBS.

The spherulitic morphology of neat PPC, neat PBS and their copolymers were studied in detail with POM and presented in Fig. 6. The average diameter of spherulites of the copolymers increases with increasing the block size of PBS segment from about 70 μm (Fig. 6B, PPC-*mb*-PBS 1200 × 1700) to 600 μm (Fig. 6E, PPC-*mb*-PBS 4000 × 4400), respectively. Moreover, the crystallinity of PPC-*mb*-PBS 4000 × 4400 is the highest than that all of PPC-*mb*-PBSs, which is comparable with neat PBS. The results are shown in Table 3. The PPC-*mb*-PBS with the block size of 2500/1700 and 3100/1700 shows no spherulitic morphology (Fig. S6†), indicating that the crystalline structure of PBS segments was restrained by PPC segments to form an amorphous structure. These results are consistent with those demonstrated in DSC measurements in second heating run.

### Improvement of mechanical strength at low temperature

**Fig. 7:**
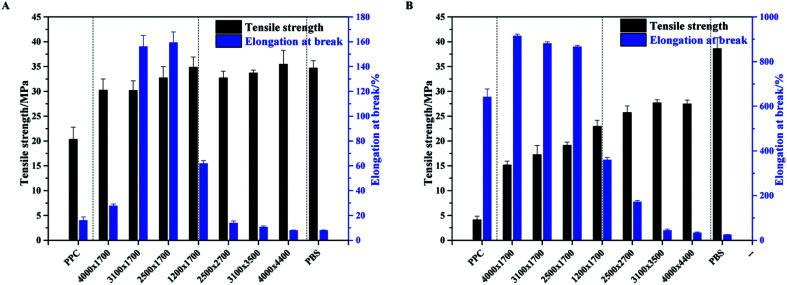
Tensile property of PPC-*mb*-PBS copolyester films at (A) 273 K, and (B) 300 K.

It is well known that PPC is an amorphous polymer with a *T*_g_ around room temperature. Therefore, it is exhibits tough and flexible natures at temperatures higher than room temperature, while rigid and brittle natures at temperatures lower than room temperature. PBS is a crystalline polymer with a melting point around 120 °C and a very low *T*_g_ of around −40 °C. Therefore, it yields high mechanical strength together with excellent toughness characteristic. In this connection, the multiblock copolymers of PPC and PBS oligomers are expected to have low brittle–toughness transition temperature and superior mechanical properties, especially low temperature property when compared with neat PPC. Fig. 7 shows the tensile properties of neat PPC, neat PBS and PPC-*mb*-PBSs. It is apparent that all the polymers exhibit higher tensile strengths than those of neat PPC at both 273 K and 300 K. The reinforcement of copolymers is believed to be the presence of crystalline PBS segment within PPC-*mb*-PBS. The crystalline domains act as strong non-covalent cross-linking domains, and greatly enhance the strength of copolymer. In addition, the tensile strength displays an increasing tendency with the decrease of PPC/PBS block ratios as well as the increase of PBS block length for a certain PPC/PBS block ratio in the multiblock copolymers. It is in consistence with the crystallinity variation tendency of the multiblock copolymers. On the other hand, due to the high content of PPC in the multiblock copolyester (PPC-*mb*-PBS 4000 × 1700, 3100 × 1700, 3500 × 1700), the PBS segments are blocked to crystallize and form amorphous phase as shown in Fig. S5.† The amorphous phase is completely miscible with PPC segment to provide larger elongation when compared with neat PPC. Particularly, these PPC-*mb*-PBSs show a higher elongation at break than neat PPC at low temperature of 273 K. A visual examination (sample deformation shown as a short movie) of tested samples is also available in ESI.†

**Fig. 8:**
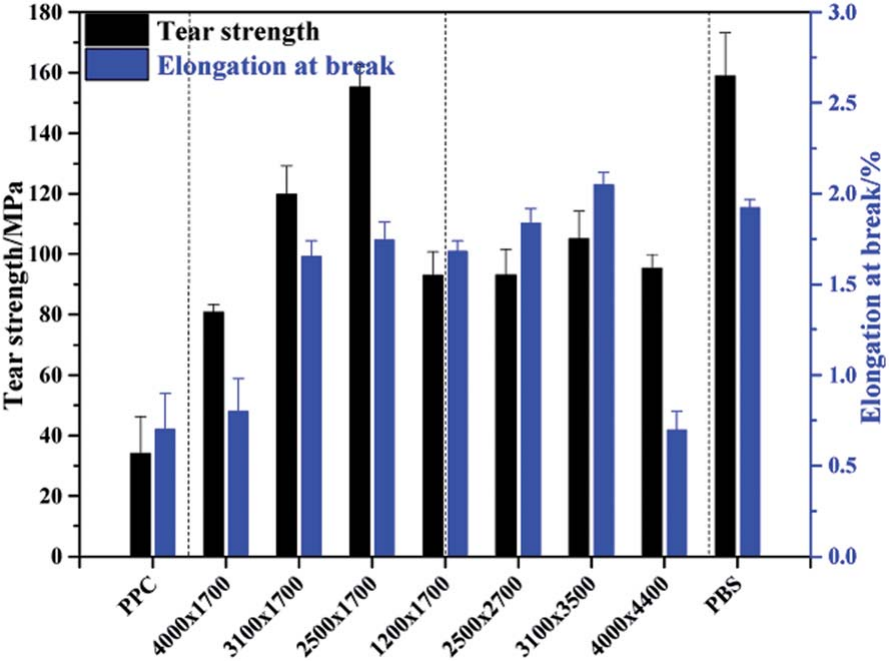
Tear strength and elongation at break of PPC-*mb*-PBS polyester films at 273 K.

For the multiblock copolymers with PPC/PBS block ratio close 1 : 1 (PPC-*mb*-PBS 1200 × 1700, 2500 × 2700, 3100 × 3500, 4000 × 4400), the average diameter of spherulites is found to be large (shown in Fig. 6). The crystallinity of PBS segment is higher than 30% and increases with increasing its block length (Table 3). Such a large spherulite and high crystallinity restricts the segmental motion of the copolymers, as a consequent, the elongation of these copolymers decreases dramatically. Moreover, low temperature tearing strength of PPC-*mb*-PBS films are investigated in detailed as shown in Fig. 8. It can be seen that PBS film exhibits much higher tearing strength and toughness than neat PPC film at 273 K. The incorporation of PBS block can dramatically improve the low temperature tearing properties of PPC. Amongst these copolymers, the PPC-*mb*-PBS 2500 × 1700 shows the best low temperature tearing property, which is comparable to neat PBS.

It should be noted that the simple blends of neat PPC and PBS fail to improve the mechanical properties of PPC by introducing PBS segments. A short video (Movie 2 in ESI†) of a tensile testing process of the PPC-*mb*-PBS 4000 × 1700 and the PPC/PBS (70/30) blend clearly shows that the blend is much brittle than the multiblock copolymer. Apparently, the covalent linked multiblock copolymer of PPC and PBS segments can fully combine their respective advantages and endows the multiblock copolymers with excellent mechanical properties, making it more potential candidate as packaging materials.

### Dynamic mechanical analysis

**Fig. 9:**
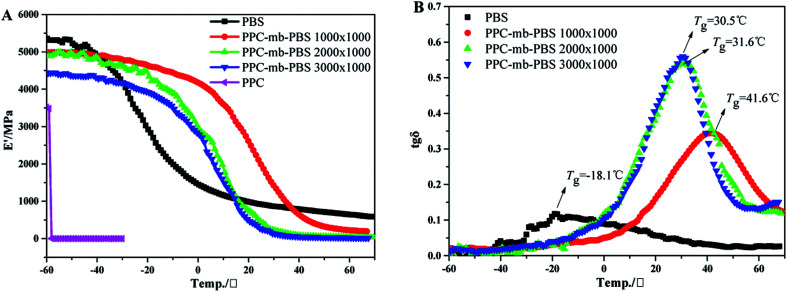
The variation of storage modulus *E*′ (A) and tan *δ* (B) with temperature for PPC, PBS and copolymers. The experiment is performed under tension mode at frequency of 1 Hz, static force of 3 N and 3 °C min^−1^ from −60 to 70 °C.

Dynamic mechanical analysis is further performed to evaluate the mechanical properties of the as-prepared multiblock copolymers at different temperatures. Storage modulus *E*′ of the copolymers is shown in Fig. 9A. Due to the brittle natures, neat PPC is broken and *E*′ value falls sharply by loading static force during the measurement. However, the *E*′ value of copolymers increases with increasing the block size of PBS segment at low temperature. Moreover, no tensile fracture was observed, indicating that PBS can not only reinforce copolymers, but also improve the flexibility of copolymers due to the presence of amorphous PBS segments. The *E*′ value of PPC-*mb*-PBS 1200 × 1700 is larger than other copolymers, such as 2500 × 1700 and 3100 × 1700 because of the presence of PBS spherulites. The results can be evidenced as POM images in Fig. 6.

It is well known that DMA is more sensitive than DSC to determine thermal transitions (such as glass transitions and other secondary transitions). Due to the sensitive limitation of DSC, the *T*_g_ from DSC curve is unchanged. In order to further confirm the conclusions from DSC, the DMA was conducted. Fig. 9B shows the tan *δ* peaks of different copolymers, and these peaks correspond to their glass transition temperature, which is consistent with the results from DSC measurements. It should be noted that only one transition temperature can be detected, which implies that there is no phase separation between PPC and PBS segments. Moreover, the *T*_g_ of PPC-*mb*-PBS with block size of 1200 × 1700, 2500 × 2700 and 3100 × 3500 is between the *T*_g_ of neat PBS and PPC.^50^ It is believed that the copolymerization between oligomeric PPC–OH and PBS–COOH results in a multiblock copolymer with very good miscibility and one glass transition temperature and endows the PPC-*mb*-PBS with low brittle–toughness transition.^50^ Consequently, the multiblock copolymer exhibits improved mechanical strength, especially the highly toughness at low temperature. The *T*_g_ determined by different method varies from the determination condition, especially the frequency. Generally, DMA is much sensitive compared with DSC to determine thermal transitions, especially for secondary transitions. The *T*_g_ measured by DMA is generally higher than that by DSC because of the frequency of DMA is much higher than DSC machine. The *T*_g_ from DSC curve is unchanged mainly due to the presence of crystalline for the copolymers.

## Conclusions

Multiblock copolymers (PPC-*mb*-PBSs) with designed sequence structures can be readily synthesized from low molecular weight and hydroxyl end-capped PPC oligomer (PPC–OH) and carboxyl end-capped PBS oligomer (PBS–COOH) *via* a coupling reaction. The crystallinity, thermal and mechanical properties of these PPC-*mb*-PBSs can be adjusted by simply changing the composition of copolymer and the block length of PPC and PBS segments. The PBS segments in multiblock copolymers endow the crystallinity that acts as physical cross-links and then reinforces the copolymer. The multiblock copolymers exhibit only one glass transition temperature at about −40 °C because of their miscible characteristic between PPC and PBS segments. The extremely low *T*_g_ endows the copolymer with excellent mechanical strength at low temperature when compared with neat PPC. The results demonstrate that the copolymerization technology between PPC and oligomeric PBS can effectively address the poor mechanical properties of PPC at low temperature, which then extending the potential and practical application.

## Conflicts of interest

There is no conflict to declare.

## Supplementary Material

RA-008-C8RA01588K-s001

RA-008-C8RA01588K-s002

RA-008-C8RA01588K-s003

## References

[cit1] Feng J., Zhuo R. X., Zhang X. Z. (2012). Prog. Polym. Sci..

[cit2] Jung J. H., Ree M., Kim H. (2006). Catal. Today.

[cit3] Naik P. U., Refes K., Sadaka F., Brachais C. H., Boni G., Couvercelle J. P., Picquet M., Plasseraud L. (2012). Polym. Chem..

[cit4] Shen Y., Xianhai Chen A., Gross R. A. (1999). Macromolecules.

[cit5] Wang X. L., Zhuo R. X., Liu L. J., He F., Liu G. (2002). J. Polym. Sci., Part A: Polym. Chem..

[cit6] Lu X. B., Shi L., Wang Y. M., Zhang R., Zhang Y. J., Peng X. J., Zhang Z. C., Li B. (2006). J. Am. Chem. Soc..

[cit7] Coates G. W., Moore D. R. (2004). Angew. Chem., Int. Ed..

[cit8] Lu X. B., Darensbourg D. J. (2012). Chem. Soc. Rev..

[cit9] Hilf J., Frey H. (2013). Macromol. Rapid Commun..

[cit10] Tullo A. H. (2011). Chem. Eng. News.

[cit11] Li G. F., Luo W. H., Xiao M., Wang S. J., Meng Y. Z. (2015). Chin. J. Polym. Sci..

[cit12] Kim I., Yi M. J., Lee K. J., Park D. W., Kim B. U., Ha C. S. (2006). Catal. Today.

[cit13] Tryznowski M., Tomczyk K., Fraś Z., Gregorowicz J., Rokicki G., Wawrzyńska E., Parzuchowski P. G. (2012). Macromolecules.

[cit14] Nagiah N., Sivagnanam U. T., Mohan R., Srinivasan N. T., Sehgal P. K. (2012). Adv. Eng. Mater..

[cit15] Li X. H., Meng Y. Z., Zhu Q., Tjong S. C. (2003). Polym. Degrad. Stab..

[cit16] Lu X. L., Zhu Q., Meng Y. Z. (2005). Polym. Degrad. Stab..

[cit17] Huang G., Zou Y., Xiao M., Wang S., Luo W., Han D., Meng Y. (2015). Polym. Degrad. Stab..

[cit18] Luinstra G. (2008). Polym. Rev..

[cit19] Gao L. J., Xiao M., Wang S. J., Meng Y. Z. (2008). J. Appl. Polym. Sci..

[cit20] Gao L. J., Du F. G., Xiao M., Wang S. J., Meng Y. Z. (2008). J. Appl. Polym. Sci..

[cit21] Seong J. E., Na S. J., Cyriac A., Kim B. W., Lee B. Y. (2010). Macromolecules.

[cit22] Wu G. P., Darensbourg D. J., Lu X. B. (2012). J. Am. Chem. Soc..

[cit23] Darensbourg D. J., Wu G. P. (2013). Angew. Chem., Int. Ed..

[cit24] Darensbourg D. J., Ulusoy M., Karroonnirum O., Poland R. R., Reibenspies J. H., Cetinkaya B. (2009). Macromolecules.

[cit25] Jeske R. C., Rowley J. M., Coates G. W. (2008). Angew. Chem., Int. Ed..

[cit26] Zhu Y., Romain C., Williams C. K. (2015). J. Am. Chem. Soc..

[cit27] Romain C., Zhu Y., Dingwall P., Paul S., Rzepa H. S., Buchard A., Williams C. K. (2016). J. Am. Chem. Soc..

[cit28] Allen S. D., Moore D. R., Lobkovsky E. B., Coates G. W. (2002). J. Am. Chem. Soc..

[cit29] Xu J. and Guo B. H., Microbial Succinic Acid, Its Polymer Poly(butylene succinate), and Applications, 2010

[cit30] Xu J., Guo B. H. (2010). Biotechnol. J..

[cit31] Sinha Ray S., Okamoto K., Okamoto M. (2003). Macromolecules.

[cit32] Li S. L., Wu F., Yang Y., Wang Y. Z., Zeng J. B. (2015). Polym. Adv. Technol..

[cit33] Zeng X., Wu B., Wu L., Hu J., Bu Z., Li B. G. (2014). Ind. Eng. Chem. Res..

[cit34] Bae Y. H., Huh K. M., Kim Y., Park K. (2000). J. Controlled Release.

[cit35] Lee J., Bae Y. H., Sohn Y. S., Jeong B. (2006). Biomacromolecules.

[cit36] Hazer B., Ayas A., Beşirli N., Saltek N., Baysal B. M. (2010). Macromol. Chem. Phys..

[cit37] Ignatious F., Lenz R. W., Kantor S. W. (1994). Macromolecules.

[cit38] Jeon O., Lee S. H., Kim S. H., Lee Y. M., Kim Y. H. (2003). Macromolecules.

[cit39] Cohn D., Salomon A. H. (2005). Biomaterials.

[cit40] Zhao Q., Cheng G., Li H., Ma X., Zhang L. (2005). Polymer.

[cit41] Pang M. Z., Qiao J. J., Jiao J., Wang S. J., Xiao M., Meng Y. Z. (2008). J. Appl. Polym. Sci..

[cit42] Chen G. J., Wang Y. Y., Wang S. J., Xiao M., Meng Y. Z. (2013). J. Appl. Polym. Sci..

[cit43] Meng Y. Z., Du L. C., Tiong S. C., Zhu Q., Hay A. S. (2002). J. Polym. Sci., Part A: Polym. Chem..

[cit44] Bakkour Y., Darcos V., Li S., Coudane J. (2012). Polym. Chem..

[cit45] Chrissafis K., Paraskevopoulos K. M., Bikiaris D. N. (2005). Thermochim. Acta.

[cit46] Darensbourg D. J., Wei S. H. (2012). Macromolecules.

[cit47] Inoue S., Tsuruta T., Takada T., Miyazaki N., Kambe M., Takaoka T. (1975). Appl. Polym. Symp..

[cit48] Liu B., Chen L., Zhang M., Yu A. (2015). Macromol. Rapid Commun..

[cit49] Varghese J. K., Na S. J., Ji H. P., Woo D., Yang I., Lee B. Y. (2010). Polym. Degrad. Stab..

[cit50] Chen G. J. , Preparation and Properties of Poly(propylene carbonate) Blend Films, Sun Yat-sen University, 2013

