# Supplementary material for: Multiblock copolymers of PPC with oligomeric PBS: with low brittle–toughness transition temperature
Source: RSC Adv. 2018 Apr 18;8(26):14722–31. doi: 10.1039/c8ra01588k (PMC9080016; doi:10.1039/c8ra01588k)
Supplement: RA-008-C8RA01588K-s003 [file RA-008-C8RA01588K-s003.pdf]

Electronic Supplementary Material (ESI) for RSC Advances.
This journal is © The Royal Society of Chemistry 2018

Supplementary material

Multiblock Copolymers of PPC with Oligomeric PBS: With Low Brittle-toughness Transition Temperature

Jiaxiang Qin, Limiao Lin, Shuanjin Wang, Shuxian Ye, Weikeng Luo, Min Xiao, Dongmei Han* and Yuezhong Meng*

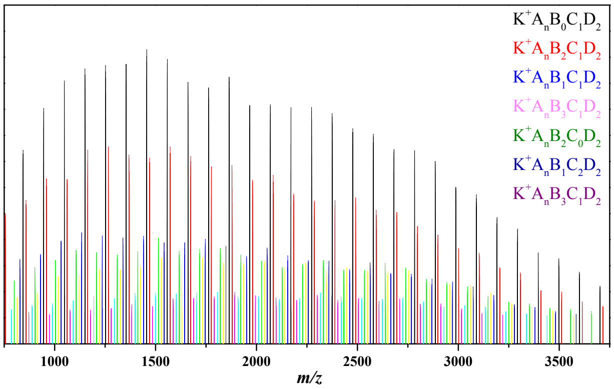

Fig. S1 MALDI-TOF mass spectrum of PPC-OH

A= -[$OCH_2CH(CH_3)OCO$]-; B=-[$OCH_2CH(CH_3)$]-; C=-[$OCH_2CH_2CH_2CH_2$]-;D=-OH+H

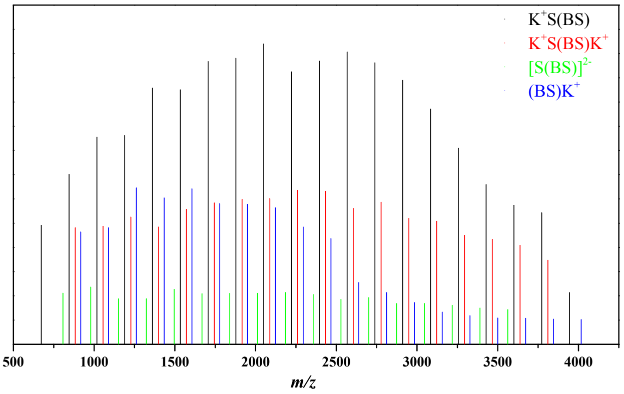

Fig. S2 MALDI-TOF mass spectrum of PBS-COOH

B= 1,4-Butanediol, S= Succinic acid

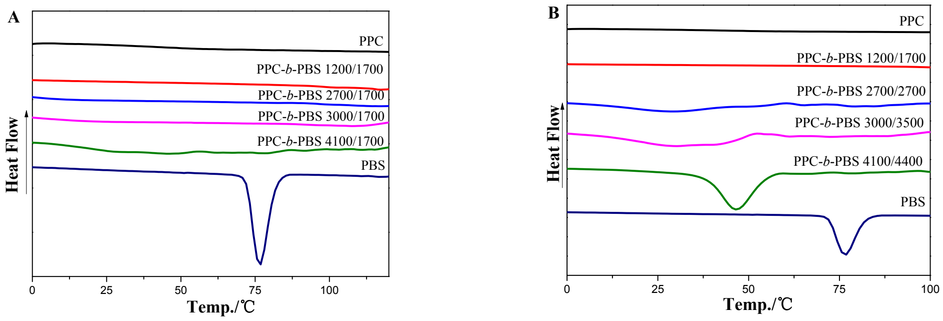

Fig. S3 Cooling run from the DSC test of (A) PPC, PBS and PPC-*mb*-PBS with the block size of 1200/1700, 2500/1700, 3100/1700, 4000/1700 and (B) PPC, PBS and PPC-*mb*-PBS with the block size of 1200/1700, 2500/2700, 3100/3500, 4000/4400

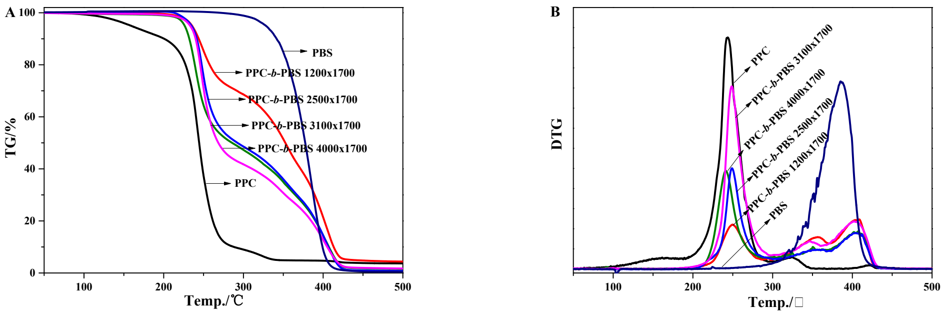

Fig. S4 TG thermograms of PPC, PBS and PPC-*mb*-PBS with the block size of 1200/1700, 2500/1700, 3100/1700 and 4000/1700: (A) TG; (B) DTG

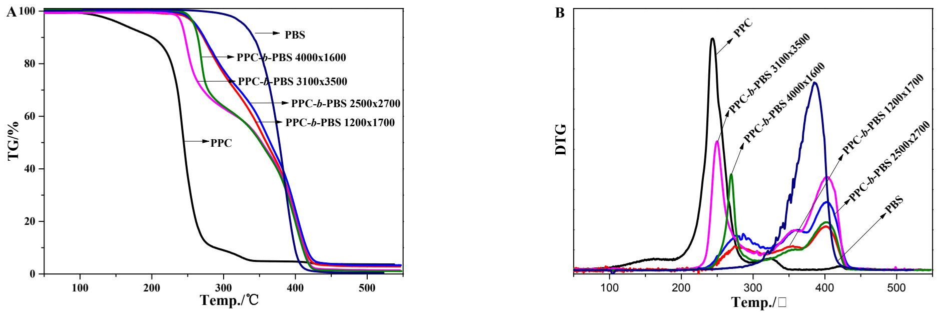

Fig. S5 TG thermograms of PPC, PBS and PPC-mb-PBS with the block size of 1200/1700, 2500/2700, 3100/3500 and 4000/4400: (A) TG; (B) DTG

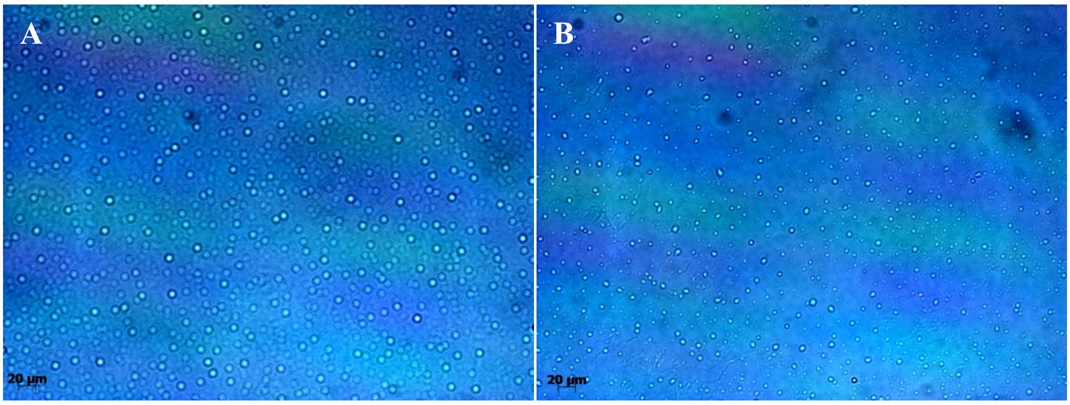

Fig. S6 Polarizing optical micrographs of PPC-mb-PBS 2500/1700 (A) and PPC-mb-PBS 3100/1700 (B) crystallized at 90 °C for 2 h.
